# Knowledge, perceptions and uptake of human papilloma virus vaccine among adolescent girls in Kampala, Uganda; a mixed-methods school-based study

**DOI:** 10.1186/s12887-023-04174-z

**Published:** 2023-07-17

**Authors:** Glet Kakuru Bitariho, Doreen Tuhebwe, Arnold Tigaiza, Aisha Nalugya, Tonny Ssekamatte, Suzanne N Kiwanuka

**Affiliations:** 1 Department of Disease control and Environmental Health, School of Public Health, College of Health Sciences, Makerere University, P.O BOX 7072, Kampala, Uganda; 2 Elevate Research and Health Services Limited, P.O BOX 3712, Kampala, Uganda; 3 Department of Health Policy Planning and Management, School of Public Health, College of Health Sciences, Makerere University, P.O BOX 7072, Kampala, Uganda

**Keywords:** Knowledge, Perceptions, Uptake, HPV vaccine, Adolescent girls, 10–14 years

## Abstract

**Background:**

Cervical cancer is a major public health challenge, accounting for substantial morbidity and mortality. Human Papilloma Virus (HPV) vaccination is the recommended primary public health intervention for HPV infection prevention. However, there’s limited evidence on the level of knowledge, attitude, and practices of adolescent girls regarding HPV vaccination in Kampala city, Uganda. This study assessed the knowledge, perceptions, and practices of adolescent girls aged 10-14 years towards HPV vaccination program in Kampala, Uganda to generate evidence to guide programs targeted at improving uptake of the vaccine.

**Methods:**

A convergent parallel mixed methods study was conducted in Kampala, Uganda. A structured questionnaire was used to elicit data from 524 adolescent girls. In addition, 6 Focus group discussions, and 24 key informant interviews (teacher and parents) were conducted. Multistage and purposive sampling techniques were used to select quantitative and qualitative participants respectively. Quantitative data were entered using epidata, cleaned and analyzed using Stata v14 while qualitative data were analyzed using thematic content analysis in atlas ti version 8.

**Results:**

Overall, only 8.6% (45/524) of the girls had completed the HPV vaccine schedule of two dozes, 49.2% (258/524) of the girls had low knowledge about the HPV vaccine and teachers and parents affirmed this lack of knowledge among adolescent girls especially concerning the target age group, dosage, and vaccine interval. About 51.9% (272/524) of girls had negative perceptions towards HPV vaccination. Parents expressed negative perceptions, beliefs, superstitions, and safety concerns of the vaccine.Girls residing in rural areas (adjusted prevalence ratio, aPR = 0.35, C. I = 0.14–0.85) had lower knowledge levels compared to those in urban areas. Girls whose mothers were healthcare providers (aPR = 1.94, C. I = 1.10–3.41), girls with high knowledge levels (aPR = 1.79, C. I = 1.21–2.63) and positive perceptions (aPR = 2.87, C. I = 1.93–4.27) had a higher prevalence of being fully vaccinated.

**Conclusion:**

Girls generally had low levels of knowledge, negative perceptions, and poor uptake of HPV vaccination. We recommend sensitization campaigns in schools and communities to improve awareness, perceptions, and practices of stakeholders towards HPV vaccination.

## Introduction

Cervical cancer is the fourth most prevalent cancer in women globally, with an estimated 570,000 new cases and 311,000 deaths in 2018 [1]. Its burden is however worse in low and middle-income countries where approximately 84–90% of its morbidity and mortality occur [2, 3]. In Uganda, evidence shows a crude incidence of 30 women per 100,000 and the age-standardized cervical cancer incidence at 56.2% per 100,000 women in 2020 [4]. Furthermore, about half of women with cervical cancer in Uganda die from it within three years of diagnosis and more than 80% within five years [4, 5]. This is attributed to the limited access to and poor uptake of preventive measures, late diagnosis of the cancer and poor access to treatment options such as surgery, radiotherapy and chemotherapy for late-stage cancers [1].

Human papillomavirus (HPV), a group of extremely common viruses is responsible for causing cervical cancer. HPV is mainly transmitted through sexual contact and the majority of cases are infected shortly after onset of sexual activity [6]. Despite its oncogenicity, HPV is preventable. WHO’s comprehensive approach to cervical cancer prevention and control recommends a set of actions and interventions across the life course. Adolescent girls aged 9 to 14 years, among other interventions, are recommended to be fully vaccinated before becoming sexually active [6]. To this regard, in November 2015, Uganda rolled out free HPV vaccination for adolescent girls 10–14 years in its immunization schedule across all districts. However, coverage of the second dose has remained as low as 41% since the introduction, way below the Ministry of health targets of 80% [4, 7]. In Kampala, Uganda’s capital city, the coverage of HPV vaccination is similarly low [7], despite the availability of several and widely distributed vaccination centers across the city. The Ministry of Health Uganda suspects the low uptake of HPV to be due to the limited knowledge and awareness of HPV and HPV vaccine in communities, confusion over eligibility for vaccination, and insufficient knowledge among stakeholders [13], but the evidence is limited.

Studies elsewhere have shown that knowledge and perceptions can predict uptake of health services including HPV vaccination, [8–10]. In a cross-sectional study conducted in Mali, HPV and cervical cancer knowledge was found to be low; only 2.0% of respondents knew that HPV is a sexually transmitted infection (STI), yet, 100% said they would be willing to receive HPV vaccination [11]. In Indonesia, 64% of women had good knowledge, 62% had positive perception of cervical cancer and HPV vaccination, and 92% tended to accept HPV vaccination [12]. Similar studies have been conducted in other parts of the world and in Uganda in different populations including women and mothers of children but little has been done among the adolescent girls themselves especially in the context of urban communities that are assumed to have access due to proximity to health facilities in Uganda. This study therefore assessed the knowledge, perceptions and uptake of the HPV vaccine among adolescent girls aged 10–14 years in Kampala City in order to generate information that could guide programing of future HPV vaccination campaigns in Kampala City and similar context.

## Materials and methods

### Study design, setting and population

Our study utilized a convergent parallel mixed-methods design for data collection. This design involved the collection and analysis of both quantitative and qualitative data, and thereafter the two were compared and integrated. This allowed us to gather a more comprehensive understanding of the factors influencing knowledge and perceptions of HPV vaccination among girls in our study population. Data collection was conducted in Kampala between February and April 2021. Kampala is the capital and largest city in Uganda with an estimated population of 1,507,114, of which 724,326 are males [14]. The city has five administrative units; the Central division, Kawempe division, Makindye division, Nakawa division, and Rubaga division. The district houses Uganda’s national referral hospitals, including Mulago national specialized hospital, Kiruddu hospital, Kawempe hospital, and Butabika national mental referral hospital and a number of high-profile schools. The study population included adolescent girls aged 10–14 years who were enrolled in schools in Kampala. The age bracket 10–14 years is the target group for HPV vaccination in Uganda [4, 15] and the vaccine is offered to all eligible girls in-and out-of school through school vaccination and community outreaches respectively.

### Sample size

The Kish Leslie (1965) formula was used to determine sample size [16]. A prevalence of 17.6% [17] was used. Considering a 95% level of confidence, an error rate (d) of 0.05, a Z score of 1.96, a 17% non-response rate and a design effect of 2 yielded a sample size of 521 adolescent girls. The data collection team however collected up to 524 interviews which were considered in the analysis. We conducted a total of 6 focus group discussions (FGDs) with adolescent girls aged 10–14 years and 24 key informant interviews (KIIs). A total of 12 KIIs were conducted among teachers, (6 were males and 6 females). In addition, 12 KIIs were conducted among parents, with 6 mothers and six fathers. The total number of KIIs and FGDs was determined by thematic saturation, i.e., the point when new data collection ceased to provide additional insights or perspectives on the research questions.

### Sampling

**Fig. 1:**
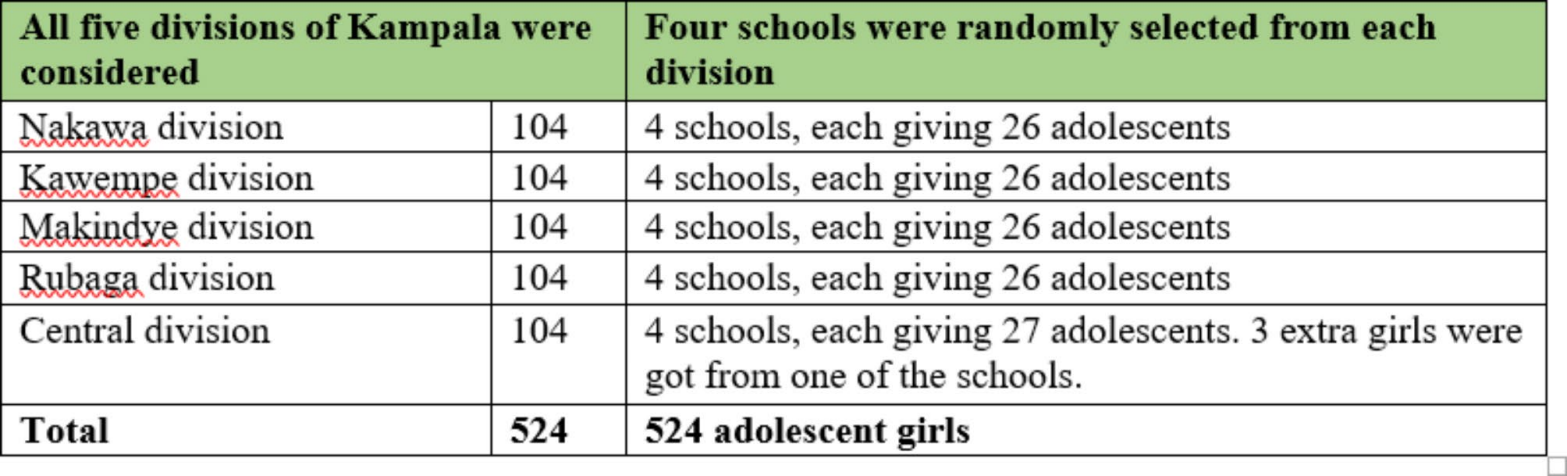
Distribution of study subjects across divisions and schools

All the five divisions in Kampala were considered. In each division we sampled 105 adolescent girls to come up with the sample size required. A list of primary schools in each division was obtained from Kampala Capital City Authority (KCCA), the urban authority body of the city. We then used random sampling to select four schools from each division’s list using the Ms Excel randomizer. This gave a total of 20 schools. The sample size of 521 was divided among the 20 schools and each was to give 26 pupils to meet the sample size as indicated in Fig. 1. Both private and public schools were included in this study to ensure the inclusion of pupils from all socio-economic strata. Each of the 20 selected schools was visited by the research team and administrative approval was obtained from the school leadership. After approval, sampling frames of students from upper primary classes (primary five, six and seven) were obtained from each school. The above classes were selected upon guidance of the teachers that these were the classes with girls who met our age inclusion criteria. At the time of data collection however, schools except candidate classes had been closed as a measure to control the spread of COVID-19, implemented by the government. Therefore, majority of study respondents were obtained from primary seven. Since 26 pupils were needed from each school, a random sample of 26 adolescent girls aged 10–14 years was selected from each class using Ms Excel randomizer. Girls below the age of 10 and above the age of 14 were excluded from the sampling frame.

Qualitative respondents were purposefully selected from each division and included the following: adolescent girls aged 10–14 years, their teachers, and parents. The teachers and parents were selected based on their knowledge and experience, level of influence and involvement in the health and well-being of the adolescent girls, and willingness to share information. Furthermore, we selected teachers based on their legal registration status at the respective schools and key roles in the girls’ lives as health advisors in the school administrative structure (i.e., senior woman and senior man teacher, head teacher, and class teacher). Adolescent girls for the FGDs were selected based on their experiences receiving or not receiving HPV vaccination and their willingness to share information. For representativeness of views and gender inclusivity, among the key informants, the study purposively engaged equal numbers of males and female teachers and parents.

### Data collection tools and techniques

A semi-structured questionnaire was used to obtain data from adolescent girls aged 10–14 years. The tool contained questions on the knowledge about the vaccination program, perceptions towards the HPV vaccine and vaccination and practices towards HPV vaccination, as adopted from the African Women Awareness of cancer (AWACAN) tool for breast and cervical cancer [18]; and modified to suit HPV. During data collection, the adolescent girls were engaged during appropriate breaks like lunch time to avoid disruption of school activities. During the engagement with each girl, the study team did self-introductions, explained the purpose of the study, and then sought informed assent before administering the questionnaire.

FGD guides and KII guides were used to explore the knowledge, perceptions, and practices of the pipuils and key informants towards the HPV vaccination program. The FGD and KII guides were developed through a process of review of existing literature on HPV vaccination and in consultation with experts in the field of reproductive health in the Ministry of Health and qualitative research methods at the Makerere University School of Public Health (MakSPH). The guides were designed to explore the key themes and topics related to knowledge, perceptions, and uptake of HPV vaccine among adolescent girls and their parents/guardians and teachers. The guides were pretested with a small group of participants to assess their clarity, relevance, and comprehensiveness before the actual data collection. The FGD and KII guides were also modified iteratively during the data collection process based on the emerging themes and issues from the previous interviews/discussions.

The research team engaged parents in the study during morning or evening hours as they dropped off and picked up pupils from school while, teachers and adolescent girls were engaged during appropriate breaks such at break tea or lunch time. After meeting the identified stakeholders, the study team did self-introductions, explained the purpose of the study, and then sought informed consent before engaging interviews. We conducted FGDs with the adolescent girls because we wanted to gather their perspectives on knowledge, perceptions, and uptake of the HPV vaccine in a group setting, where they could interact and share their experiences with one another. Each FGD consisted of 7–12 adolescent girls. This allowed for a more comprehensive understanding of their attitudes and beliefs towards the vaccine. On the other hand, we conducted key informant interviews (KIIs) with the adults because they were seen as more knowledgeable and experienced about the vaccination process and the factors that influence uptake. Additionally, it was easier to schedule interviews with the adults than to arrange for them to attend group discussions, especially considering their busy schedules. All qualitative interviews were moderated by a female well-trained research assistant with vast experience in qualitative research and work with young adolescent girls. The moderator was assisted by a note taker to ensure no information was missed out. The surveys lasted approximately 30 min, the KIIs 40 min while the FGDs took about an hour.

### Study variables and measurement

The main outcome variables included; the level of knowledge, perceptions and uptake of HPV vaccination among adolescent girls. Knowledge was measured using a set of 8 questions. For each question, a correct response was awarded a score of 1 and any wrong response was awarded 0. This made the total score 8 in case a respondent passed all the question and 0 if they failed all. The knowledge score ranged between 0 (minimum mark observed) to 8 (maximum mark observed). A respondent’s score therefore depended on how many questions they passed. The mean score was 4.05(SD: 2.41), the median score was 5 and range (8 − 0) was 8. The median score (5) was taken as a cut-off point. Those who obtained a score ≥ 5 were considered as knowledgeable and those who scored below the median score were considered as having a low level of knowledge. The same approach had been applied in other studies to assess the level of knowledge on HPV vaccination [12, 19].

A 3-point Likert scale (agree, neither agree nor disagree/neutral, disagree) was used to measure perceptions of girls on HPV vaccination using a set of 13 statements. For each question, a desired perception was awarded a score of 1 and the undesired perception and neutral was awarded a zero. The minimum score observed was 0 and the maximum score was 13. The perceptions score therefore ranged from 0 to 13 depending on how the respondent agreed or disagreed to the statements. The mean score was 7.83(SD: 3.01), the median score was 8 and the range (13 − 0) was 13. The median score (8) was then taken as a cut-off point and those who obtained a score **≥** 8 were considered as having positive perceptions and those who scored below 8 were considered as having a negative perception.

Conversely, uptake of the HPV vaccine was measured by asking the girls if they have ever received the HPV vaccine. Girls responded Yes or No depending on whether they have ever been vaccinated against HPV or not and a follow up question on number of dozes received was asked.

The independent variables included socio-demographic factors such as; age, religion, education level, parents’ occupation, and sex debut. Age was collected as a continuous variable and later categorized into (10–12 and 13–14) years. Education level was categorized into primary five, six, and seven. The education levels in Uganda include pre-primary, primary, secondary and post-secondary. Primary level has seven classes, (primary one to seven), each class running for a period of one year. The official school going age bracket for pre-primary level is 3–5 years; 6–12 years for primary level, 13–18 years for secondary level and 19–24 years for post-secondary school level. However, due to the impact of the COVID-19 pandemic to the education system, we were able to recruit girls aged 13–14 at primary level [20]. Religion was grouped into Catholic, Anglican, Muslim, Pentecostal, and Others. The residence area was categorized into urban and peri-urban, and father’s occupation and mother’s occupation were categorized as doctor/healthcare provider, public employed, privately employed, self-employed, and retired/unemployed. We did not categorize healthcare providers amongst publicly employed because we thought these could have a significant impact on the uptake of vaccination among the girls. Lastly, sex debut was asked as “ever had sex” with responses yes/no.

### Quality control and assurance

Both qualitative and quantitative tools were translated to Luganda and the researchers ensured to only recruit research assistants who were conversant with Luganda since it was the commonest language spoken by the prospective respondents. Research assistants with a good command of English were recruited to conduct interviews, however, the interviews were conducted in a language most comfortable to the respondent. Research assistants were trained on the research protocol and ethical issues surrounding the study. To ensure the appropriateness of the questions, reliable and accurate information, pre-visits to the study setting and pretesting of the study tools were done. The tool was pre-tested from schools in Wakiso district, an equally urban area with similar characteristics as those in the sampling frame in Kampala. Furthermore, the research assistants were supervised by a team led by the Principal Investigator during the actual data collection exercise. The supervisors ensured that the tool is checked and field edited, if necessary, to ensure completeness of data before data entry.

### Data management and analysis

Quantitative data was field edited for consistency and omissions. Data materials were secured under lock and key and were only accessed by the study team. The data was entered using Epidata, edited for consistency, and then exported to STATA 14 for statistical analysis. Descriptive analysis was done to generate the mean and the standard deviation for continuous variables and proportions for categorical variables. Frequency tables were used to present these results. The modified Poisson regression model was used for further analysis to establish the associations between the independent and outcome variables at both bivariate and multivariable analysis. Modified Poisson regression has been shown to provide more accurate estimates of the strength of the association in situations where the outcome is common (greater than 10%) [21–24]. At the multivariable level, variables with a p-value less than 0.2 were included in the model. To assess the goodness of fit of the modified Poisson regression model, we calculated the log-likelihood. The log-likelihood is a measure of how well the model predicts the observed data, and is calculated as the sum of the logarithms of the probability densities of the observed data under the fitted model. We calculated the log-likelihood for the modified Poisson regression model using the “log likelihood” output from the “glm” command in Stata. The log-likelihood was used to compare the fit of different models, such as comparing nested models or models with different predictor variables. We also used the Akaike information criterion (AIC) and Bayesian information criterion (BIC), which incorporate the log-likelihood and the number of parameters in the model, to compare the fit of different models and select the best-fitting model. A p-value of less than 0.05 was considered statistically significant and we present the prevalence ratios. All results were summarized in tables.

All qualitative interviews were digitally recorded with permission from respondents and transcribed verbatim. The transcripts were proofread before importing them into a qualitative data management software- atlas.ti6. Data coding and analysis were conducted subsequently. An initial codebook using a sample of transcripts was developed. The developed codebook was then applied to the entire atlas project with any emerging codes being added in the process. Thematic analysis was used and results were presented using themes with typical quotations from different qualitative interviews to summarize knowledge and perceptions. Content analysis was used for practices and these were summarized using a matrix.

## Results

### Socio-demographic characteristics of the respondents

A total of 524 girls were interviewed. The mean age of the respondents was 12.9 years (SD ± 0.8). Close to two-thirds, 63.9% (335/524) of the respondents were aged 13–14 years, 97.1% (509/524) were in primary seven. and 95.8% (502/524) were residing in an urban area. Close to a third, 31.7% (166/524) of the respondents were catholic, and less than a tenth, 7.1% (37/524) had ever had sexual intercourse (Table 1).

**Table 1:** Socio-demographic characteristics of the respondents

Variable	Description	Frequency (n = 524)	Percentage (%)
**Age (in years) (mean = 12.9, S. D = ± 0.84)**	10–12	189	36.1
	13–14	335	63.9
**Class**	Primary five	4	0.8
	Primary six	11	2.1
	Primary seven	509	97.1
**Religion**	Catholic	166	31.7
	Anglican	111	21.2
	Muslim	100	19.1
	Pentecostal	87	16.6
	Others (specify)	60	11.5
**Residence area**	Urban	502	95.8
	Peri-urban	22	4.2
**Father’s occupation**	Doctor/healthcare provider	27	5.2
	Public employed	97	18.5
	Private employed	121	23.1
	Self-employed	266	50.8
	Retired/unemployed	13	2.5
**Mother’s occupation**	Doctor/healthcare provider	27	5.2
	Public employed	57	10.9
	Private employed	83	15.8
	Self-employed	310	59.2
	Retired/unemployed	47	9.0
**Ever had sexual intercourse**	No	487	92.9
	Yes	37	7.1

### Knowledge and awareness of HPV vaccination

Almost half, 49.2% (258/524) of the respondents had low levels of knowledge on HPV vaccination, 70.6% (370/524) were aware of the HPV vaccine, 66.6% (349/524) believed that the HPV vaccine reduced the risk of cervical cancer, and 68.7% knew the target age group for the HPV vaccine. More than half, 54.8% (287/524) knew the recommended number of HPV vaccine doses and 52.9% (277/524) did not know the correct interval between the HPV vaccines doses. About 17.0% (89/524) of the respondents wrongfully mentioned that the vaccine is only for sexually active people (Table 2).

**Table 2:** Knowledge and awareness of HPV vaccination among adolescent girls in Kampala City, Uganda

Variable	Description	Frequency (n = 524)	Percentage (%)
There is a vaccine that protects against HPV	Yes	370	70.6
	No	31	5.9
	I don’t know	123	23.5
The HPV vaccine reduces chances of getting cervical cancer	Yes	349	66.6
	No	31	5.9
	I don’t know	144	27.5
Once vaccinated, women no longer have to be screened for cervical cancer	Yes	148	28.2
	No	147	28.1
	I don’t know	229	43.7
The target age group for the HPV vaccine in Uganda	10 years and above	360	68.7
	I don’t know	164	31.3
The HPV vaccine is only for sexually active people	Yes	89	17.0
	No	227	43.3
	I don’t know	208	39.7
The HPV vaccine is most effective if given to people who have never had sex	Yes	134	25.6
	No	125	23.9
	I don’t know	265	50.6
Number of HPV vaccine doses given in Uganda	Two doses	287	54.8
	I don’t know	237	45.2
Interval between HPV vaccine doses	6 months	247	47.1
	I don’t know	277	52.9
Level of knowledge	High knowledge level	266	50.8
	Low knowledge level.	258	49.2

Some key stakeholders also expressed during key informant interviews that they were not sure of some of the information related to the vaccine, including the target age group and recommended number of doses and also believed the girls may not know.

“*I don’t remember properly but I think they are either 3 or 4 or 2 doses. I don’t know. When they (health workers) came here last time, they took girls for the first dose then they came for the second dose. I don’t know if that was all. I am not sure whether 3 or 4, they did not tell us or the girls. So I don’t think the girls know*” (Key informant, teacher)

“*Well, those nurses who came said they wanted children of 14 years and in some cases, they said they wanted people who were still producing. So, I don’t know who is recommended but I think sexually active women are recommended.*” (Key informant, teacher)

However, in the FGDs with adolescent girls, some respondents were knowledgeable about the vaccine, target population for the vaccine, when it’s preferable to receive the vaccine, number of doses and interval between the doses. A girl expressed that;

“*You can receive it (HPV vaccine) when you are about 9 to 12 years. It is preferred that you receive the vaccine when you are a virgin. It is given before sexual contact. The vaccine is given to females and males, and they are given two doses. The second interval is after six months after the first interval*.” (Participant 1, FGD Adolescent girls)

### Factors associated with the level of knowledge regarding HPV vaccination

At a multivariable analysis, area of residence and religion were significantly associated with level of knowledge of girls on HPV vaccination. The prevalence of a high knowledge level was 0.38 times higher among respondents who were residing in a peri-urban area (aPR = 0.38, C. I = 0.14–0.85, P -value = 0.028) compared to those living urban areas. Furthermore, the prevalence of a high knowledge level was 38% higher among respondents from the Pentecostal faith (aPR = 1.38, C. I = 1.11–1.71, P -value = 0.004) compared to those from the catholic faith (Table 3).

**Table 3:** Factors associated with the level of knowledge of HPV vaccination among adolescent girls in Kampala City, Uganda

Variable	Level of knowledge		PR at 95% CI	aPR at 95% CI	P-values
	High (n = 266)	Low (n = 258)			
	%	%			
**Age**					
10–12	50.8	49.2	1		
13–14	50.8	49.3	1.00 (0.84–1.19)		
**Class**					
Primary five	75.0	25.0	1	1	
Primary six	90.9	9.1	1.21 (0.67–2.20)	1.20 (0.84–1.39)	0.542
Primary seven	49.7	50.3	0.67 (0.37–1.18)	0.69 (0.79–1.48)	0.183
**Religion**					
Catholic	47.6	32.4	1	1	
Anglican	56.8	43.2	1.19 (0.95–1.50)	1.21 (0.99–1.51)	0.097
Muslim	49.0	51.0	1.03 (0.80–1.33)	1.06 (0.71–1.71)	0.657
Pentecostal	65.5	34.5	1.34 (1.10–1.72)	1.38 (1.11–1.71)	0.004*
Others (specify)	30.0	70.0	0.63 (0.42–0.96)	0.66 (0.44-1.00)	0.052
**Residence area**					
Urban	52.2	47.8	1	1	
Peri-urban	18.2	81.8	0.35 (0.14–0.85)	0.38 (0.16–0.90)	0.028*
**Father’s occupation**					
Healthcare provider	59.3	40.7	1		
Public employed	56.7	43.3	0.96 (0.67–1.37)		
Private employed	54.6	45.5	0.92 (0.65–1.31)		
Self-employed	45.9	54.1	0.77 (0.55–1.09)		
Retired/unemployed	53.9	46.2	0.91 (0.50–1.64)		
**Mother’s occupation**					
Healthcare provider	55.6	44.4	1		
Public employed	54.4	45.6	0.98 (0.65–1.48)		
Private employed	50.6	49.4	0.91 (0.61–1.36)		
Self-employed	49.0	50.9	0.88 (0.62–1.26)		
Retired/unemployed	55.3	44.7	1.00 (0.65–1.52)		
**Ever had sexual intercourse**					
No	50.7	49.3	1		
Yes	51.4	48.7	1.01 (0.73–1.40)		

* ***Considering a 95% CI, a p-value ≤ 0.05 was considered to be statistically significant***

***PR = prevalence ratio aPR = adjusted prevalence ratio***

### Perceptions of HPV vaccination

Slightly more than half, 51.9% (272/524) had negative perceptions towards HPV vaccination.

Only 49.1% (257/524) agreed that the HPV vaccine would be beneficial to a teenage girl or boy’s future health, and 36.3% (190/524) disagreed that HPV vaccination is not necessary because a Pap test can be done to rule out cervical cancer. About 27.9% (146/524) of the girls agreed that if other people knew they received the HPV vaccine, they would be embarrassed and 17.4% (91/524) believed that it would be difficult and embarrassing for them to ask for the HPV vaccine because it is associated with a sexually transmitted infection. Close to three quarters, 72.7% (381/524) agreed that they would be willing to recommend the HPV vaccine to others and 80.5% (422/524) agreed that they would be willing to receive the HPV vaccine if offered for free (Table 4).

**Table 4:** Perception of HPV vaccination among adolescent girls in Kampala City, Uganda

Variable	Description	Frequency (n = 524)	Percentage (%)
Getting the HPV vaccine would be beneficial to a teenage girl or boy’s future health	Agree	257	49.1
	Neutral	140	26.7
	Disagree	127	24.2
HPV vaccination is not necessary because a Pap test can be done to rule out cervical cancer	Agree	93	17.8
	Neutral	241	46.0
	Disagree	190	36.3
I believe it would be difficult and embarrassing for me to ask for the HPV vaccine because it is associated with a sexually transmitted infection	Agree	91	17.4
	Neutral	123	23.5
	Disagree	310	59.2
If other people knew I received the HPV vaccine, I would be embarrassed	Agree	146	27.9
	Neutral	75	14.3
	Disagree	303	57.8
Knowing the risks of HPV, I intend on taking the HPV vaccine in the future	Agree	361	68.9
	Neutral	94	17.9
	Disagree	69	13.2
The HPV vaccine is effective in preventing cervical cancer	Agree	282	53.8
	Neutral	144	27.5
	Disagree	98	18.7
I will take the vaccine because I feel at risk of getting HPV	Agree	370	70.6
	Neutral	103	19.7
	Disagree	51	9.7
I feel the vaccine will keep me safe from cervical Cancer	Agree	400	76.3
	Neutral	91	17.4
	Disagree	33	6.3
I feel it is better to be vaccinated before becoming sexually active	Agree	390	74.4
	Neutral	87	16.6
	Disagree	47	9.0
I feel only sexually active girls should get the vaccine	Agree	146	27.9
	Neutral	108	20.6
	Disagree	270	51.5
HPV vaccine may have long negative effects on me	Agree	122	23.3
	Neutral	234	44.7
	Disagree	168	32.1
I would be willing to recommend the HPV vaccine to others	Agree	381	72.7
	Neutral	80	15.3
	Disagree	63	12.0
I would be willing to receive the HPV vaccination if offered for free?	Agree	422	80.5
	Neutral	57	10.9
	Disagree	45	8.6
If no/disagree why?	Possible side effects	19	18.6
	Low awareness of HPV	30	29.4
	Not being at risk	30	29.4
	Cost	3	2.9
	Others (specify)	20	19.6
Perception of girls	Positive perception	252	48.1
	Negative perception	272	51.9

Negative perceptions toward HPV vaccination were also recorded especially among parents and these are passed down to the girls. A respondent was quoted expressing that some parents are against vaccinating their children due to the beliefs that the vaccine will make the children barren/infertile. Furthermore, some respondents believed that the government was aiming at wiping their children out through HPV vaccination.

“*They (Parents) have got all sorts of beliefs originating from people out there. People tell parents that, “you people, your children are going to be barren because of the vaccine.” So, somebody thinks about that and has to pick between cancer and infertility for their children. When girls are drawn into all this, it affects their perceptions too*” (Key informant, teacher)

“*Yeah, usually there are superstitions within the community and among girls that the government does not want the girls to produce many children*” (Key informant, Parent)

“*Some parents are against the vaccine because of their political views. Some are anti-government and think that whatever program the government brings could be detrimental to their children’s health.*” (Key informant, teacher)

“*For me I wanted but when I brought the letter home, (consent form) and gave it to my parents, my mother told me that I should not go to school that day; that she talked with the teacher on my behalf.*” (Participant 5, FGD Adolescent girl)

### Factors associated with perceptions toward HPV vaccination

The prevalence of positive perceptions towards HPV vaccination was found to be 3.02 times (aPR = 3.02, C. I = 2.37–3.84, *P* -value = 0.000) higher among girls with a high knowledge level compared to those with a low level of knowledge (Table 5).

**Table 5:** Factors associated with perception of HPV vaccination among adolescent girls in Kampala City, Uganda

Variable	HPV perceptions		PR at 95% CI	aPR at 95% CI	P-values
	Positive (n = 252)	Negative (n = 272)			
	%	%			
**Age**					
10–12	16.6	53.4	1		
13–14	49.0	51.0	1.05 (0.87–1.26)		
**Class**					
Primary five	75.0	25.0	1		
Primary six	90.9	9.1	1.21 (0.67–2.20)	0.94 (0.37–2.36)	0.891
Primary seven	47.0	53.1	0.63 (0.35–1.11)	0.74 (0.30–1.85)	0.519
**Religion**					
Catholic	44.6	55.4	1		
Anglican	54.1	46.0	1.21 (0.95–1.54)	1.11 (0.90–1.38)	0.333
Muslim	48.0	52.0	1.08 (0.83–1.40)	1.09 (0.85–1.39)	0.511
Pentecostal	59.8	40.2	1.34 (1.05–1.70)	1.15 (0.94–1.41)	0.175
Others (specify)	30.0	70.0	0.67 (0.44–1.03)	0.86 (0.59–1.24)	0.425
**Residence area**					
Urban	49.0	51.0	1		
Peri-urban	27.3	72.7	0.56 (0.28–1.11)	0.84 (0.42–1.67)	0.621
**Father’s occupation**					
Healthcare provider	59.3	40.7	1	1	
Public employed	47.4	52.6	0.80 (0.55–1.17)	0.84 (0.60–1.18)	0.315
Private employed	53.7	46.3	0.91 (0.64–1.29)	0.95 (0.70–1.27)	0.713
Self-employed	45.1	54.9	0.76 (0.54–1.07)	0.90 (0.68–1.20)	0.474
Retired/unemployed	38.5	61.5	0.65 (0.30–1.38)	0.71 (0.39–1.30)	0.271
**Mother’s occupation**					
Healthcare provider	59.3	40.7	0.85 (0.55–1.32)		
Public employed	47.4	52.6	1		
Private employed	53.7	46.3	1.08 (0.74–1.59)		
Self-employed	45.1	54.9	0.80 (0.56–1.15)		
Retired/unemployed	38.5	61.5	0.84 (0.53–1.33)		
**Ever had sexual intercourse**					
No	49.1	50.9	1		
Yes	35.1	64.9	0.72 (0.46–1.12)	0.71 (0.46–1.10)	0.125
**Knowledge level**					
Low knowledge level	22.9	77.1	1		
High knowledge level	72.6	27.4	3.17 (2.51–4.02)	3.02 (2.37–3.84)	0.000*

* ***Considering a 95% CI, a p-value ≤ 0.05 was considered to be statistically significant***

***PR = prevalence ratio aPR = adjusted prevalence ratio***

### HPV vaccination status

Close to three quarters of the girls, 72.5% (380/524) had not received a single shot of HPV vaccine, 27.5% (144/524) had ever received the HPV vaccine and only 8.6% (45/524) had completed the vaccination schedule of two dozes. Majority of the vaccinated girls 84.7% (122/144) received their first shot while in the age range of 10 to 12 years. The most mentioned reasons for failure to receive the vaccine was not being aware of the HPV vaccination, 48.2% (183/524) and where to access the vaccine, 28.4% (108/524) (Table 6).

**Table 6:** HPV vaccination status of adolescent girls in Kampala City, Uganda

Variable	Description	Frequency (n = 524)	Percentage
Ever received the HPV vaccine	Yes	144	27.5
	No	380	72.5
Number of HPV vaccine doses received	0	380	72.5
	1	99	18.9
	2 or more	45	8.6
Completed HPV vaccination schedule	Yes	45	8.6
	No	479	91.4
Age at which the respondent got the first shot (in years) **(n = 144) (mean = 11.3, S.D = 1.1**)	10–12	122	84.7
	13–14	22	15.3
Reasons for not receiving the vaccine (n = 380)	I am not aware of HPV vaccination	183	48.2
	I don’t know where to access the vaccine	108	28.4
	I don’t have time	11	2.9
	I don’t see the need	11	3.2
	Fear of side effects	25	6.6
	Others	41	10.8

Parents also expressed that they had not supported their daughters to go for HPV vaccination mainly because of fear of side effects. In contrast, teachers mentioned that in support of HPV vaccination program, they provide information on HPV, encourage children to get immunized, and support healthcare workers in whichever way they could.

“*We organize the venue for vaccination in school, encourage children to get immunized and allow health workers to vaccinate children. We also feed them, in most cases when they come, they take breakfast here*. *We support their activities.*” (Key informant, Teacher)

“*I haven’t taken her for HPV vaccination. We’ve all been reluctant to do so because we have concerns about the side effects*” (key informant, Parent)

### Factors associated with HPV vaccination status

At multivariable level, girls’ knowledge level, perceptions regarding HPV, and their mothers’ occupation were significantly associated with receiving the HPV vaccine. The prevalence of HPV vaccination was 94% higher among girls whose mothers were healthcare providers by profession (aPR = 1.94, C. I = 1.10–3.41, P -value = 0.021) compared to those whose mothers were civil servants. Girls with high level of knowledge on HPV vaccination (aPR = 1.79, C. I = 1.21–2.63, P -value = 0.003) reported a higher prevalence of HPV vaccination compared to those with a low knowledge level. Girls with positive perceptions toward HPV vaccination (aPR = 2.87, C. I = 1.93–4.27, P -value = 0.000) had a higher prevalence of HPV vaccination compared to those with negative perceptions (Table 7).

**Table 7:** Factors associated with HPV vaccination among adolescent girls

Variable	HPV vaccination status		PR at 95% CI	aPR at 95% CI	P-values
	Yes (n = 144)	No (n = 380)			
	%	%			
**Age**					
10–12	29.1	70.9	1		
13–14	26.6	73.4	0.91 (0.69–1.21)		
**Class**					
Primary five	25.0	75.0	1	1	
Primary six	72.7	27.3	2.90 (0.51–16.53)	0.94 (0.37–2.36)	0.891
Primary seven	26.5	73.5	1.06 (0.19–5.84)	0.74 (0.30–1.85)	0.519
**Residence area**					
Urban	28.5	71.5	1		
Peri-urban	4.6	95.5	0.16 (0.02–1.09)	0.26 (0.041-1.60)	0.145
**Father’s occupation**					
Doctor/healthcare provider	48.2	91.3	1	1	
Public employed	33.0	51.9	0.69 (0.42–1.11)	1.14 (0.73–1.80)	0.561
Private employed	27.3	67.0	0.57 (0.35–0.92)	0.87 (0.59–1.29)	0.493
Self-employed	23.3	72.7	0.48 (0.31–0.76)	0.53 (0.31–1.05)	0.096
Retired/unemployed	30.8	76.7	0.64 (0.26–1.58)	1.00 (0.49–2.03)	0.997
**Mother’s occupation**					
Public employed	24.6	75.4	1	1	
Doctor/healthcare provider	51.9	48.2	2.11 (1.18–3.78)	1.94 (1.10–3.41)	0.021*
Private employed	22.9	77.1	0.93 (0.51–1.70)	0.89 (0.49–1.62)	0.712
Self-employed	25.5	74.5	1.04 (0.63–1.70)	1.21 (0.75–1.96)	0.433
Retired/unemployed	38.3	61.7	1.56 (0.87–2.79)	1.55 (0.92–2.62)	0.102
**Ever had sexual intercourse**					
No	28.1	71.9	1		
Yes	18.9	81.1	0.67 (0.34–1.33)	0.71 (0.46–1.10)	0.125
**Knowledge level**					
Low level of knowledge	12.8	87.2	1		
High knowledge level	41.7	58.3	3.26 (2.30–4.63)	1.79 (1.21–2.63)	0.003*
**Perceptions**					
Negative	11.4	88.6	1		
Positive	44.8	55.2	3.93 (2.75–5.63)	2.87 (1.93–4.27)	P ≤ 0.001*

* ***Considering a 95% CI, a p-value ≤ 0.05 was considered to be statistically significant***

***PR = prevalence ratio aPR = adjusted prevalence ratio***

Respondents further affirmed that knowledge regarding HPV vaccination among the different key stakeholders, including parents and teachers is really low due to a lack of sensitization on the matter and it could be affecting uptake of the vaccines. In this regard, key informants were quoted saying;

“*Some schools seek for parental consent before giving the vaccine and some parents also fear to give the consent because they are not aware of what would come next (side effects)*” (key informant, Teacher)

“*Sensitization is lacking. People are not aware that this vaccine is available and the healthcare facilities that offer this vaccine are not very common. You find the health centers or medical facilities that have those vaccines are fewer, which might affect uptake. People think that they are supposed to pay some money to access the vaccine and they don’t know that it’s free of charge. It’s now up to the government to do some bit of sensitization. You find that most parents are illiterate. The illiterates may not understand the benefits, so we need more sensitization about it.*” (Key informant, Parent)

## Discussion

This study aimed to assess stakeholders’ knowledge, perceptions, and practices towards the HPV vaccination program in Kampala. The findings revealed that 49.2% of the girls had low knowledge of HPV vaccination. Girls residing in peri-urban areas had lower knowledge levels compared to those in urban areas. About 52% of girls had negative perceptions towards HPV vaccination and only 8.6% (45/524) of the adolescent girls had received both doses of the vaccine. Girls whose mothers were healthcare providers, girls with high knowledge levels and those with positive perceptions had a higher prevalence of those vaccinated with at least one doze. Qualitative findings supported the quantitative analyses.

Almost half (49.2%) of the girls had low levels of knowledge regarding HPV vaccination. Similarly, a knowledge gap was revealed among other key stakeholders (including, teachers and parents) on the causes and risk factors for HPV and HPV vaccine. A low knowledge level among parents and teachers is likely to impact the girls’ knowledge, attitudes, and practices concerning HPV vaccination. These findings concur with those in a study conducted in Lira district which showed that adolescent girls had poor knowledge levels (56.1%) [17]. Other studies have also indicated that knowledge on HPV vaccination among teachers and parents is lacking [25, 26]. The knowledge gap reported in our study could be attributed to the lack or limited sensitization opportunities on HPV vaccination, as reported by some of the key informants in this study. Therefore, this study highlights the need for comprehensive health education and community outreach programs that cover HPV and HPV vaccination to increase the awareness among the different stakeholders.

After adjusting for confounders, girls residing in peri-urban areas were 0.4 times less likely to have a high level of knowledge compared to those in urban areas. This could be because girls in urban areas are exposed to various sources of information which could be lacking or limited in rural areas. A review of the literature has supported these findings. For instance, a study conducted by Chen, Orom [27] revealed that compared to urban residents, rural residents had lower access to health information from sources including primary care providers, specialist doctors, blogs, and magazines, and less use of search engines. This could therefore explain the lower knowledge level among girls residing in peri-urban areas. These findings highlight the need for increasing access to information among girls in rural areas.

Regarding perceptions toward HPV vaccination, this study found that 51.9% of the girls had negative perceptions towards HPV vaccination. The negative perceptions could be explained by the lack of knowledge and limited sensitization among the girls as highlighted by the key informants in this study, hence contributing to the negative perceptions. These findings corroborate those in another cross-sectional study which revealed uncertainties about the need for the vaccine among the girls in terms of perceived risk, and safety concerns of the vaccine [28]. This, therefore, implies that there is a need for strategies targeted at raising awareness of adolescent girls on HPV, and consequently improving their perceptions.

Similarly, other stakeholders portrayed negative perceptions towards HPV vaccination. The study found negative beliefs, superstitions, and safety concerns among parents, which are likely to influence their willingness to get their daughters vaccinated. As indicated in the literature, knowledge level can greatly impact individuals’ perceptions. Therefore, the negative beliefs portrayed in this study could be due to a lack of awareness among these stakeholders as remarked by some of the key informants. The study findings concur with those in other cross-sectional studies which also found that parents and teachers had negative attitudes toward HPV and the vaccine [29, 30].

Girls with a high level of knowledge regarding HPV were more likely to have positive perceptions compared to those with a low level of knowledge. Knowledge has been shown to predict individuals’ attitudes toward a behavior. This could be because an increase in the level of knowledge leads to a better understanding of the inherent risks related to HPV and cervical cancer, which consequently translates into positive attitudes towards the uptake of the vaccine. Therefore, change of perceptions towards the uptake of the HPV vaccine may require continuous sensitizations.

The study estimated the level of uptake of HPV vaccination (receiving two doses of the vaccine) at 8.6%, which is significantly way below the targeted coverage of 80% [31]. This low uptake has also been reported in a study conducted in Northern Uganda [17] and in a multi-level analysis of the 2016 Uganda Demographic and Health Survey conducted by Isabirye, Mbonye [32]. The low HPV vaccination uptake could be attributed to the low knowledge levels regarding the HPV vaccine as indicated in our study. Similarly, girls with a higher knowledge showed higher prevalence of being fully vaccinated. This could be because girls with a higher knowledge level are likely to have a better understanding of their risk for HPV and the benefits of the HPV vaccine, and consequently have higher prevalence of uptake of the vaccine as opposed to the less knowledgeable. These findings concur with those in a cross-sectional study conducted in Greece by Donadiki, Jiménez-García [10], which showed that knowledge level was a predictor of vaccination uptake among adolescent girls. Furthermore, the most mentioned reason in this study for failure to receive the vaccine was not being aware of the HPV vaccination. The, key informants in the study also cited lack of awareness, sensitization and negative parents’ beliefs as barriers to uptake of HPV vaccination.

From the qualitative findings, teachers exhibited good practices toward HPV vaccination. They expressed supporting healthcare workers in HPV vaccination through availing information to the girls and parents to enhance their willingness to take up the vaccine. In contrast, the study revealed poor practices among parents concerning vaccination. The difference in practices among teachers and parents could be because teachers are exposed to health-related information while at school which could lead to a better understanding of the need for vaccination and consequently influence their practices [25].

The study also indicated that the prevalence of HPV vaccination were higher among girls with positive perception towards the HPV vaccine, and girls whose mothers were healthcare providers by profession. The positive perceptions including, perceived susceptibility to HPV, the severity of cervical cancer, benefits, and efficacy of the HPV vaccine could motivate the girls to take up the vaccine. Such girls would therefore have better practices concerning HPV vaccination. However, these findings do not corroborate those in a study conducted in England which found no significant correlation between attitude and HPV vaccination uptake [33].

### Strengths and limitations of the study

This study reached a large number (524 adolescent girls aged 10–14 years) which makes the findings more reliable and generalizable. In addition, the study included a qualitative component which further triangulated the quantitative findings. The practices were however assessed based on self-report and self-reported responses are prone to recall bias and social desirability bias. In our case, we did not ask to look at the vaccination cards of the adolescents and therefore could not ascertain whether the girls were actually vaccinated or not. We however minimized the bias in responding by emphasizing the importance of the study and the need for them to answer questions correctly. Our sampling strategy involved selection of all five divisions in Kampala and each division producing 104 students to come up with the sample size required. This may have affected representativeness of HPV vaccine uptake. However, given that all the five divisions have government hospitals where HPV services including vaccination are given for free, we believe that this sampling strategy is representative of Kampala City. Additionally, this was a cross-sectional study design and is limited in its ability to establish temporality between the exposure and outcome variables. There’s a need for more rigorous studies with longitudinal designs to better understand the relationships between HPV knowledge, attitudes, and vaccine uptake.

## Conclusion

Adolescent girls in this study had relatively low levels of knowledge about the HPV vaccine, more than half had negative perceptions towards the vaccine, and less than a tenth had completed the vaccination schedule. The low uptake was attributed to lack of awareness on HPV vaccination and where to access the vaccines. This indicates a critical need for awareness raising about HPV vaccines among all stakeholders. Girls residing in urban areas had better knowledge levels compared to those in peri-urban areas. Girls with high knowledge levels had positive perceptions towards vaccination and positive perceptions were associated with uptake of at least one doze of the HPV vaccine. Qualitative findings further affirmed low knowledge levels, negative perceptions, and beliefs about the vaccine among adolescents and parents.

## Data Availability

The data used for this manuscript is available from the corresponding author on reasonable request.

## Abbreviations

AWACAN: African Women Awareness of Cancer
DHIS: District Health Information System
EPI: Expanded program for immunization
FGD: Focus group discussion
HPV: Human Papilloma Virus
IRB: Institutional Review Board
KAP: Knowledge, Attitudes, and Perceptions.
KCCA: Kampala City Council Authority
KI: Key Informant Interviews
MOH: Ministry of Health
PATH: Program for Appropriate Technology in Health
REC: Research and Ethics Committee
UNEPI: Uganda National Expanded Program for Immunization
UBOS: Uganda Bureau of Statistics
WHO: World Health Organization
